# Accuracy of methods for diagnosing heart diseases in cats

**DOI:** 10.14202/vetworld.2020.872-878

**Published:** 2020-05-09

**Authors:** Tanarut Laudhittirut, Natrada Rujivipat, Kornnicha Saringkarisate, Peeraya Soponpattana, Teerawat Tunwichai, Sirilak Disatian Surachetpong

**Affiliations:** Department of Veterinary Medicine, Faculty of Veterinary Science, Chulalongkorn University, Bangkok 10330, Thailand

**Keywords:** cardiac disease diagnosis, N-terminal Pro-B-type natriuretic peptide, thoracic radiography, ultrasonography

## Abstract

**Aim:**

This study aimed to determine the accuracy of the current methods for diagnosing heart diseases in cats.

**Materials and Methods:**

The data of 58 cats were retrospectively retrieved. Cats were classified into two groups: Thirty-eight cats with heart diseases and 20 healthy cats. Echocardiography was the gold standard method for diagnosing heart disease. The results of seven methods were retrieved: (1) Vertebral heart score (VHS) with a cutoff value >8, (2) VHS with a cutoff value >8.5, (3) multiplication of cardiac length (L) and width (W), (4) multiplication of cardiac L and W divided by the L of the fourth sternal thoracic bone, (5) N-terminal Pro-B-type natriuretic peptide (NT-proBNP) point-of-care test, (6) subjective ultrasonographic assessment of the left atrial size, and (7) subjective radiographic assessment of the left atrial size. Cross-tabulation was used to calculate the sensitivity, specificity, accuracy, positive predictive value, and negative predictive value for each test. This study found that using the NT-proBNP point-of-care test was optimal in the diagnosis of cats with heart disease.

**Results:**

The subjective ultrasonographic assessment of the left atrial size was good for diagnosing hypertrophic cardiomyopathy and congestive heart failure.

**Conclusion:**

This study showed that the more tests used, the higher the reliability of the diagnosis.

## Introduction

Heart disease is common in cats, affecting 10%-15% of all cats [1,2]. Most cats affected by heart disease may not show clinical signs. A few cats may develop signs of congestive heart failure (CHF) or suddenly die [3]. Common heart diseases in cats include cardiomyopathies, such as hypertrophic cardiomyopathy (HCM), restrictive cardiomyopathy (RCM), and unclassified cardiomyopathy (UCM). Congenital cardiac disorders in cats, such as atrioventricular valve dysplasia and septal defects, are less common [4].

Echocardiography is the gold standard method for diagnosing heart disease in cats. Despite being a definitive method, performing echocardiography requires experienced sonographers. Furthermore, the required equipment is expensive. Therefore, echocardiography is difficult to access and unavailable in primary care animal hospitals in some regions. At present, there are three common methods for diagnosing heart diseases in cats: Radiography, thoracic focused assessment with sonography for trauma (TFAST), and cardiac biomarker measurements. These methods may help in diagnosing heart disease in cats, particularly in some locations where comprehensive echocardiography is unavailable. Radiography is the most common method used to evaluate cardiac size and shape. However, there is a limitation in using radiography in diagnosing heart disease in cats. Some types of heart disease in cats, such as cardiomyopathies, may not cause cardiac enlargement that can be detected on radiography. The VHS, a method in evaluating the size of the heart on radiography, may not distinguish cats with and without heart disease. The cutoff value of VHS for cats with heart disease is eight. Cats with a VHS >9.3 have an extremely high tendency of having heart disease. However, cats with VHS 8-9.3 are classified into a grey zone and need further investigation for the diagnosis of heart disease [5]. A new method for detecting heart diseases in cats using thoracic radiography has been published [6]. This method has 82.2% sensitivity and 66.9% specificity in differentiating cardiac and noncardiac diseases in cats. TFAST can also be used. This technique is a reliable method in assessing cardiac structural changes without the technical challenge and long time. Left atrial enlargement can be detected using TFAST. A left atrial enlargement is highly suggestive of a restrictive filling pattern, which is an indicator in the diagnosis of heart diseases in cats [7]. The measurement of cardiac biomarkers is another method in diagnosing heart disease in cats. Pro-B-type natriuretic peptide (proBNP) is a substance that is released from cardiac muscles when the heart is stretched or stressed [8]. At present, an N-terminal proBNP (NT-proBNP) point-of-care test kit is available. However, the test is semiquantitative and has some limitations. Noncardiac diseases, such as severe respiratory or kidney diseases (with creatinine level >2.8 mg/d), increase the likelihood of false-positive results [9]. An increase in NT-proBNP levels can be observed in cats with severe arrhythmias, pulmonary hypertension, systemic hypertension, and hyperthyroidism [10,11].

This study aimed to determine the accuracy of current methods for diagnosing heart diseases, HCM, and CHF in cats.

## Materials and Methods

### Ethical approval

Ethical approval is not necessary because of the retrospective design of the study.

### The data of cats

The medical records of cats presented to our university, from 2016 to 2018 were retrospectively reviewed. The data of cats that had undergone complete cardiac examinations, including clinical evaluations, cardiac biomarker measurements, radiography, and echocardiography, were collected. The data from cats with concurrent diseases affecting NT-proBNP levels, including hyperthyroidism, systemic hypertension, kidney diseases (creatinine level >2.8 mg/dL), and respiratory diseases, were excluded from the study. Echocardiography was used as the gold standard method for diagnosing cardiac diseases.

Digital thoracic radiographs were archived. Radiographs with pleural effusion, pneumothorax, and unclear cardiac silhouette were excluded from the study. The length (L) of the long axis (distance from the ventral border of the carina to the cardiac apex) and width (W) (maximum cardiac width perpendicular to the measurement of the long axis) of the heart were measured on the right lateral view. Five individuals performed each measurement. The interobserver coefficient of variation was calculated for each measurement method. The VHS was evaluated. Two measurement methods from a previous study [6] were performed:

1. Multiplication of L and W (L×W) with the cutoff value >16.46 cm^2^.
2. Multiplication of L and W divided by the length of the fourth sternal thoracic bone (S4) (L×W/S4) with a cutoff value >12.85 cm.

The result of a feline NT-proBNP point-of-care test kit (SNAP^®^ Feline proBNP, IDEXX, Maine, United States) was recorded as positive or negative. All results were interpreted using the automated test kit reader machine (SNAP^®^ Pro Analyzer, IDEXX, Maine, United States).

The result of the left atrial enlargement subjectively assessed by TFAST was recorded as normal or enlarged size.

Echocardiography was performed using an ultrasound machine (EKO7, Samsung Medison, Seoul, South Korea) with 2-4 and 4-12 multifrequency MHz phased array transducers. The definitive heart disease diagnosed by echocardiography was recorded. The left atrial size measured from the right parasternal cross-sectional view at the level of the left atrium was noted. A left atrial size >16 mm was classified as enlargement [7]. The diagnostic criteria for HCM included increased wall thickness (≥6 mm) and decreased left ventricular chamber size [11]. RCM was identified by left atrial or biatrial enlargement, normal left ventricular wall thickness, normal or decreased systolic function, and restrictive ventricular filling pattern with pulsed-wave Doppler echocardiography [12]. Congenital heart disease was classified according to a previously published study [13]. Ventricular septal defects were diagnosed using two-dimensional (2D) and color Doppler echocardiography to identify flow across the lesion. Mitral and tricuspid valve dysplasias were diagnosed based on the presence of abnormalities of the valve apparatus and valvular regurgitation on 2D and color Doppler echocardiography.

### Statistical analysis

Statistical analysis was performed using a commercial statistical program (SPSS software Version 22, IBM Inc., NY, USA) and cross-tabulation to determine the specificity, sensitivity, and accuracy of each test. The positive predictive value (PPV) and negative predictive value (NPV) were also calculated.

## Results

The data and radiographs of 58 cats were retrieved. Twenty-nine cats were male. Breeds of the cats included Domestic Shorthair (n=32), Persian (n=15), African Shorthair (n=4), Scottish Fold (n=2), Sphinx (n=1), Siamese (n=1), Khao Manee (n=1), Maine Coon (n=1), and British Shorthair (n=1). Twenty cats (ten males and ten females) were normal. Thirty-eight cats (twenty males and 18 females) had heart diseases, including HCM (n=24), RCM (n=5), UCM (n=3), hypertrophic obstructive cardiomyopathy (n=3), atrial septal defect (n=1), mitral valve dysplasia (n=1), and tricuspid valve dysplasia (n=1).

Seven diagnostic methods were performed in all cats: VHS at cutoff values >8 and >8.5, multiplication of the length and width of the heart (L×W), ratio of the multiplication of the length and width of the heart to the length of the fourth sternal thoracic bone (L×W/S4), subjective radiographic assessment of left atrial size (AX), NT-proBNP point-of-care test (NT-proBNP), and subjective ultrasonographic assessment of the left atrial size (AU). Interobserver variability of VHS, L×W, and L×W/S4 were 6.63%, 6.95%, and 5.66%, respectively.

The specificity, sensitivity, accuracy, PPV, and NPV of each diagnostic test and combination tests were analyzed using the diagnostic result from echocardiography as the gold standard method. The accuracy of the tests to distinguish cats with and without heart diseases, cats with and without HCM, and cats with and without CHF is summarized in Tables-1-3, respectively.

**Table-1:** Specificity, sensitivity, accuracy, positive predictive, and negative predictive values for each test in cats with (n=38) and without heart disease (n=20).

Parameters	Number positive/negative	Sensitivity (%)	Specificity (%)	Accuracy (%)	PPV	NPV
One test						
VHS>8	24/34	44.74	65.00	51.72	70.83	38.24
VHS>8.5	13/45	26.32	85.00	46.55	76.92	37.78
L×W	24/34	63.16	50.00	58.62	70.59	41.67
L×W/S4	25/33	42.10	55	46.55	64	33.33
AX	29/29	60.53	70.00	44.65	79.31	48.28
NT-proBNP	30/28	68.42	80.00	72.41	86.67	57.14
AU	16/42	42.10	100	62.07	100	47.62
Two tests						
VHS>8+L×W	18/18	56.52	61.54	58.33	72.22	44.44
VHS>8+L×W/S4	15/24	40.74	66.67	48.72	73.33	33.33
VHS>8+AX	24/19	53.57	73.33	60.47	78.95	45.83
VHS>8+NT-proBNP	15/19	61.9	84.62	70.59	86.67	57.89
VHS>8+AU	11/29	40.74	100	60	100	44.83
VHS>8.5+L×W	11/22	40	76.92	54.55	72.73	45.45
VHS>8.5+L×W/S4	9/28	25	78.57	44.74	66.67	37.93
VHS>8.5+AX	13/29	40	82.35	57.14	76.92	48.28
VHS>8.5+NT-proBNP	9/24	44.44	93.33	66.67	88.89	58.33
VHS>8.5+AU	7/36	26.92	100	55.81	100	47.22
L×W+AX	21/15	69.57	64.29	67.57	76.19	56.25
L×W+NT-proBNP	18/12	77.27	87.5	80	94.44	58.33
L×W+AU	15/23	53.57	100	65.79	100	43.48
L×W/S4+AX	17/21	52	69.23	57.89	76.47	42.85
L×W/S4+NT-proBNP	14/17	60	81.82	67.74	85.71	52.94
L×W/S4+AU	12/29	40	100	56.1	100	37.93
AX+NT-proBNP	19/19	70.83	85.71	76.32	89.47	63.16
AX+AU	12/25	52.17	100	70.27	100	56
AU+NT-proBNP	13/25	59.09	100	76.32	100	64
Three tests						
VHS>8+L×W+AX	15/13	66.67	63.64	65.52	75	53.85
VHS>8+L×W+NT-proBNP	12/9	78.57	85.71	80.95	91.67	66.67
VHS>8+L×W+AU	11/17	55	100	67.86	100	47.06
VHS>8+L×W/S4+AX	13/18	47.62	70	54.84	76.92	38.89
VHS>8+L×W/S4+NT-proBNP	11/12	66.67	87.5	73.91	90.91	58.33
VHS>8+L×W/S4+AU	10/22	43.48	100	58.06	100	38.1
VHS>8+AX+NT-proBNP	13/16	66.67	90.91	75.86	92.31	62.5
VHS>8+AX+AU	10/21	50	100	67.74	100	52.38
VHS>8+AU+NT-proBNP	10/17	62.5	100	77.78	100	64.71
VHS>8.5+L×W+AX	9/16	53.33	75	62.96	72.73	56.25
VHS>8.5+L×W+NT-proBNP	8/11	63.64	87.5	73.68	87.5	63.64
VHS>8.5+L×W+AU	7/21	38.89	100	60.71	100	47.62
VHS>8.5+L×W/S4+AX	8/21	33.33	75	50	66.67	42.86
VHS>8.5+L×W/S4+NT-proBNP	7/14	50	90	68.18	85.71	60
VHS>8.5+L×W/S4+AU	6/26	28.57	100	53.13	100	42.31
VHS>8.5+AX+NT-proBNP	9/19	53.33	92.31	71.43	88.89	63.16
VHS>8.5+AX+AU	7/25	38.89	100	65.63	100	56
VHS>8.5+AU+NT-proBNP	7/21	50	100	75	100	66.67
L×W+AX+NT-proBNP	14/10	81.25	87.5	83.33	92.86	70
L×W+AX+AU	12/15	66.67	100	77.78	100	60
L×W+AU+NT-proBNP	12/12	76.47	100	83.33	100	63.64
L×W/S4+AX+NT-proBNP	13/12	68.75	77.78	72	84.62	58.33
L×W/S4+AX+AU	11/18	55	100	68.97	100	50
L×W/S4+AU+NT-proBNP	11/16	58.82	100	73.08	100	56.25
AX+AU+NT-proBNP	10/17	62.5	100	78.57	100	66.67

AX=The subjective radiographic assessment of the left atrial size, AU=The ultrasonographic measurement of the left atrial diameter, L×W=The multiplication of the L and the W of the heart, L×W/S4=The ratio of the multiplication of the L and the W of the heart to the L of the fourth sternal thoracic bone, NPV=Negative predictive value, NT-proBNP=The NT-proBNP point-of-care test, PPV=Positive predictive value, VHS>8=Vertebral heart score at a cutoff value of >8, VHS>8.5=Vertebral heart score at a cutoff value of >8.5

**Table-2:** Specificity, sensitivity, accuracy, positive predictive, and negative predictive values of tests for distinguishing cats with (n=16) and without hypertrophic cardiomyopathy (n=42).

Parameters	Number positive/negative	Sensitivity (%)	Specificity (%)	Accuracy (%)	PPV	NPV
One test						
VHS>8	24/34	62.50	66.67	65.52	41.67	82.35
VHS>8.5	13/45	43.75	85.71	74.14	53.85	80
L×W	24/34	62.50	42.86	48.28	29.41	75
L×W/S4	25/33	56.25	61.90	60.34	36	78.79
AX	29/29	87.50	64.28	70.69	48.28	93.1
NT-proBNP	30/28	87.50	61.90	68.97	46.67	92.86
AU	16/42	62.50	85.71	79.31	62.5	85.71
Two tests						
VHS>8+L×W	18/18	66.67	58.33	61.11	44.44	77.78
VHS>8+L×W/S4	15/24	63.64	71.43	69.23	46.67	83.33
VHS>8+AX	24/19	83.33	70.97	74.42	52.63	91.67
VHS>8+NT-proBNP	15/19	90	75	79.41	60	94.74
VHS>8+AU	11/29	66.6	89.29	82.5	72.73	86.21
VHS>8.5+L×W	11/22	54.55	77.27	69.7	54.55	77.27
VHS>8.5+L×W/S4	9/28	50	85.71	76.32	55.56	82.76
VHS>8.5+AX	13/29	77.78	81.82	80.95	53.85	93.1
VHS>8.5+NT-proBNP	9/24	77.78	91.67	87.88	77.78	91.67
VHS>8.5+AU	7/36	54.55	96.88	86.05	85.71	86.11
L×W+AX	21/15	83.33	56	64.86	47.62	87.5
L×W+NT-proBNP	18/12	100	54.55	66.67	44.44	100
L×W+AU	15/23	62.5	77.27	71.05	62.5	77.27
L×W/S4+AX	17/21	81.82	70.37	73.68	52.94	90.48
L×W/S4+NT-proBNP	14/17	100	70.83	77.42	50	100
L×W/S4+AU	12/29	60	88.46	78.05	75	79.31
AX+NT-proBNP	19/19	100	70.37	78.95	57.89	100
AX+AU	12/25	83.33	92	89.19	83.33	92
AU+NT-proBNP	13/25	100	83.33	86.84	61.54	100
Three tests						
VHS>8+L×W+AX	15/13	80	57.89	65.52	50	84.62
VHS>8+L×W+NT-proBNP	12/9	100	64.29	76.19	58.33	100
VHS>8+L×W+AU	11/17	66.67	81.25	75	72.73	76.47
VHS>8+L×W/S4+AX	13/18	77.78	72.73	74.19	53.85	88.89
VHS>8+L×W/S4+NT-proBNP	11/12	100	70.59	78.26	54.55	100
VHS>8+L×W/S4+AU	10/22	63.64	85	77.42	70	80.95
VHS>8+AX+NT-proBNP	13/16	100	80	86.21	69.23	100
VHS>8+AX+AU	10/21	80	90.48	87.1	80	90.48
VHS>8+AU+NT-proBNP	10/17	100	85	88.89	70	100
VHS>8.5+L×W+AX	9/16	75	73.68	74.07	54.55	87.5
VHS>8.5+L×W+NT-proBNP	8/11	100	84.62	89.47	75	100
VHS>8.5+L×W+AU	7/21	54.55	94.12	78.57	85.71	76.19
VHS>8.5+L×W/S4+AX	8/21	71.43	82.61	80	55.56	90.48
VHS>8.5+L×W/S4+NT-proBNP	7/14	100	88.24	90.91	71.43	100
VHS>8.5+L×W/S4+AU	6/26	50	95.45	81.25	83.33	80.77
VHS>8.5+AX+NT-proBNP	9/19	100	90.48	92.86	77.78	100
VHS>8.5+AX+AU	7/25	75	95.83	90.63	85.71	92
VHS>8.5+AU+NT-proBNP	7/21	100	95.45	96.43	85.71	100
L×W+AX+NT-proBNP	14/10	100	62.5	75	57.14	100
L×W+AX+AU	12/15	83.33	86.67	85.19	83.33	86.67
L×W+AU+NT-proBNP	12/12	100	68.75	79.17	61.54	100
L×W/S4+AX+NT-proBNP	13/12	100	66.67	76	53.85	100
L×W/S4+AX+AU	11/18	81.82	88.89	86.21	81.82	88.89
L×W/S4+AU+NT-proBNP	11/16	100	84.21	88.46	70	100
AX+AU+NT-proBNP	10/17	100	90	92.86	80	100

AX=The subjective radiographic assessment of the left atrial size, AU=The ultrasonographic measurement of the left atrial diameter, L×W=The multiplication of the L and the W of the heart, L×W/S4=The ratio of the multiplication of the L and the W of the heart to the L of the fourth sternal thoracic bone, NPV=Negative predictive value, NT-proBNP=The NT-proBNP point-of-care test, PPV=Positive predictive value, VHS>8=Vertebral heart score at a cutoff value of >8, VHS>8.5=Vertebral heart score at a cutoff value of >8.5

**Table-3:** Specificity, sensitivity, accuracy, positive predictive, and negative predictive values of tests for distinguishing cats with (n=22) and without congestive heart failure (n=36).

Parameters	Number positive/negative	Sensitivity (%)	Specificity (%)	Accuracy (%)	PPV	NPV
One test						
VHS>8	24/34	63.63	72.22	68.97	58.33	76.47
VHS>8.5	13/45	40.90	88.89	70.69	69.23	71.11
L×W	24/34	63.63	44.44	51.72	41.18	66.67
L×W/S4	25/33	50.00	61.11	56.89	44	66.67
AX	29/29	72.72	63.89	67.24	55.17	79.31
NT-proBNP	30/28	81.81	66.67	72.41	60	85.71
AU	16/42	59.10	91.67	79.31	81.25	78.57
Two tests						
VHS>8+L×W	18/18	68.75	65	66.67	61.11	72.22
VHS>8+L×W/S4	15/24	58.82	77.27	69.23	66.67	70.83
VHS>8+AX	24/19	75	74.07	74.42	63.16	83.33
VHS>8+NT-proBNP	15/19	91.67	81.82	85.29	73.33	94.74
VHS>8+AU	11/29	69.23	92.59	85	81.82	86.21
VHS>8.5+L×W	11/22	53.33	83.33	69.7	72.73	68.18
VHS>8.5+L×W/S4	9/28	43.75	90.91	71.05	77.78	68.97
VHS>8.5+AX	13/29	60	85.19	76.19	69.23	79.31
VHS>8.5+NT-proBNP	9/24	77.78	91.67	87.88	77.78	91.67
VHS>8.5+AU	7/36	50	96.77	83.72	85.71	83.33
L×W+AX	21/15	78.57	56.52	64.86	52.38	81.25
L×W+NT-proBNP	18/12	81.25	64.29	73.33	72.22	75
L×W+AU	15/23	63.16	84.21	73.68	80	69.57
L×W/S4+AX	17/21	66.67	69.57	68.42	58.82	76.19
L×W/S4+NT-proBNP	14/17	76.92	77.78	77.42	71.43	82.35
L×W/S4+AU	12/29	56.25	88	75.61	75	75.86
AX+NT-proBNP	19/19	86.67	73.91	78.95	68.42	89.47
AX+AU	12/25	81.82	88.46	86.49	75	92
AU+NT-proBNP	13/25	80	95.65	89.47	92.31	88
Three tests						
VHS>8+L×W+AX	15/13	83.33	64.71	72.41	62.5	84.62
VHS>8+L×W+NT-proBNP	12/9	90.91	80	85.71	83.33	88.89
VHS>8+L×W+AU	11/17	69.23	86.67	78.57	81.82	76.47
VHS>8+L×W/S4+AX	13/18	69.23	77.78	74.19	69.23	77.78
VHS>8+L×W/S4+NT-proBNP	11/12	90	84.62	86.96	81.82	91.67
VHS>8+L×W/S4+AU	10/22	66.67	89.47	80.65	80	80.95
VHS>8+AX+NT-proBNP	13/16	90.91	83.33	86.21	76.92	93.75
VHS>8+AX+AU	10/21	88.89	90.91	90.32	80	95.24
VHS>8+AU+NT-proBNP	10/17	100	94.44	96.3	90	100
VHS>8.5+L×W+AX	9/16	72.73	81.25	77.78	72.73	81.25
VHS>8.5+L×W+NT-proBNP	8/11	77.78	90	84.21	87.5	81.82
VHS>8.5+L×W+AU	7/21	50	93.75	75	85.71	71.43
VHS>8.5+L×W/S4+AX	8/21	58.33	88.89	76.67	77.78	76.19
VHS>8.5+L×W/S4+NT-proBNP	7/14	75	92.86	86.36	85.71	86.67
VHS>8.5+L×W/S4+AU	6/26	45.45	95.24	78.13	83.33	76.92
VHS>8.5+AX+NT-proBNP	9/19	77.78	89.47	85.71	77.78	89.47
VHS>8.5+AX+AU	7/25	75	95.83	90.63	85.71	92
VHS>8.5+AU+NT-proBNP	7/21	85.71	95.24	92.86	85.71	95.24
L×W+AX+NT-proBNP	14/10	83.33	66.67	75	71.43	80
L×W+AX+AU	12/15	81.82	81.25	81.48	75	86.67
L×W+AU+NT-proBNP	12/12	85.71	90	87.5	92.31	81.82
L×W/S4+AX+NT-proBNP	13/12	81.82	71.43	76	69.23	83.33
L×W/S4+AX+AU	11/18	80	84.21	82.76	72.73	88.89
L×W/S4+AU+NT-proBNP	11/16	81.82	93.33	88.46	90	87.5
AX+AU+NT-proBNP	10/17	90	94.44	92.86	90	94.44

AX=The subjective radiographic assessment of the left atrial size, AU=The ultrasonographic measurement of the left atrial diameter, L×W=The multiplication of the L and the W of the heart, L×W/S4=The ratio of the multiplication of the L and the W of the heart to the L of the fourth sternal thoracic bone, NPV=Negative predictive value, NT-proBNP=The NT-proBNP point-of-care test, PPV=Positive predictive value, VHS>8=Vertebral heart score at a cutoff value of >8, VHS>8.5=Vertebral heart score at a cutoff value of>8.5

The best method for diagnosing cats with any heart disease was the NT-proBNP point-of-care test. Using the NT-proBNP point-of-care test in combination with the multiplication of the length and width of the heart assessed by radiography provided a higher percentage of accuracy. Using three tests together, including the NT-proBNP point-of-care test, multiplication of the length and width of the heart, and subjective radiographic assessment of the left atrial size or subjective ultrasonographic assessment of left atrial size, provided the best accuracy for diagnosing heart disease in cats.

The subjective ultrasonographic assessment of the left atrial size was the best method for diagnosing cats with HCM. A more accurate result was obtained when conducting the subjective ultrasonographic assessment of the left atrial size together with the NT-proBNP point-of-care test. The best tests for diagnosing HCM comprised the VHS at a cutoff value >8.5, subjective ultrasonographic assessment of the left atrial size, and NT-proBNP point-of-care test. This set of tests provided 96.43% accuracy.

The subjective ultrasonographic assessment of the left atrial size provided the highest percentage of accuracy. A higher percentage of accuracy was found when using a combination of tests, including the subjective ultrasonographic assessment of the left atrial size and NT-proBNP point-of-care test. Based on the results of this study, the best method to diagnose CHF in cats is to conduct a combination of tests, including VHS at a cutoff value >8, subjective ultrasonographic assessment of the left atrial size, and NT-proBNP point-of-care test.

## Discussion

This study recommends the NT-proBNP point-of-care test or subjective ultrasonographic assessment of the left atrial size as the optimum methods for diagnosing cats with heart diseases, HCM, or CHF, particularly when comprehensive echocardiography is unavailable. The previous studies suggested that the NT-proBNP point-of-care test can be used to assess the severity of heart disease and HCM in cats [14,15]. Moreover, the NT-proBNP point-of-care test may be used as a complementary test to evaluate the risk of death [16] and distinguish respiratory distress with cardiogenic and non-cardiogenic causes in cats [9,17].

The usefulness of NT-proBNP in the diagnosis of heart disease in cats has been reviewed [18]. The use of the NT-proBNP point-of-care test has some limitations: The blood sampling protocol requires centrifugation, separation, and storage of plasma within a limited time after sample collection [15]. A previous study showed that a negative result of the NT-proBNP point-of-care test could not exclude the presence of underlying heart disease [19]. In contrast, performing the subjective ultrasonographic assessment of the left atrial size is less stressful for cats than using the NT-proBNP point-of-care test because a lower degree of restraint is required [20].

The assessment of heart size on radiographs may not be suitable as a sole method for diagnosing heart diseases in cats. All radiographic assessment methods used in this study, including VHS, multiplication of the length and width of the heart, and subjective radiographic assessment of the left atrial size provided moderate sensitivity and specificity in diagnosing heart diseases, HCM, and CHF in cats. In some regions, animal hospitals generally perform thoracic radiography to diagnose heart disease in cats. However, some conditions may make interpretation of the radiographs more challenging [5,21], such as concurrent respiratory disease, heartworm disease, presence of non-cardiogenic pleural effusion, radiographic positioning, pericardial fat, and the phase of the respiratory and cardiac cycles [22]. In addition, the left atrial enlargement assessed by thoracic radiography may be absent in acute CHF in some cats [23]. For a more accurate diagnosis of heart disease in cats, we recommend using radiography in combination with other methods, such as NT-proBNP point-of-care test and subjective ultrasonographic assessment of the left atrial size.

Other tests that can help in the diagnosis of HCM in cats have been proposed, including genetic tests, other biomarkers such as cardiac troponin I, and cardiac arrhythmia test [24]. The mutation of myosin-binding protein C (MYBPC3) has been found in Maine Coon and Ragdoll cats affected with HCM [25,26]. However, not all cats with MYBP3 mutation have HCM. Moreover, some cats with MYBPC3 mutation may not develop HCM [27]. Cardiac troponin I is one of the cardiac biomarkers that may be used in test for the diagnosis of HCM with high sensitivity (91.7%) and specificity (95.4%) [28]. Plasma growth differentiation factors 8 and 11, biomarkers associated with cardiac hypertrophy in humans, have been studied in cats. However, both growth differentiation factors 8 and 11 cannot be used to differentiate normal cats and cats with HCM [29]. A previous study showed that ventricular arrhythmias are common in cats with HCM, suggesting that arrhythmia may be used as a complementary method for diagnosing heart disease in cats [30].

The limitations of the study were the small number of cats included. In addition, the use of radiographic measurement results and interpretation from five individuals rather than one may have affected the accuracy of radiography.

## Conclusion

The results of this study show that the more tests used, the more reliable the diagnosis of cats with heart disease, HCM, and CHF. However, using a combination of methods has some limitations, including the availability and cost of each test. Selecting which tests to perform depends on the purpose of diagnosis. All methods used in this study are intended for use as a first step diagnosis; therefore, echocardiography is still needed to confirm the type of heart disease in cats.

## Authors’ Contributions

TL, NR, KS, PS and TT : data collection, data analysis, and writing the first draft; NR: SDS: supervision, data validation, writing review, and editing. All authors read and approved the final manuscript.
